# Mediating effect of depression on the association between cardiovascular disease and the risk of all‐cause mortality: NHANES in 2005−2018

**DOI:** 10.1002/clc.24103

**Published:** 2023-08-18

**Authors:** Xinxin Ma, Huan Zhang, Yuan Tian, Yaping Wang, Ling Liu, Lei Wang

**Affiliations:** ^1^ Department of Psychology and Psychiatry The Second Affiliated Hospital of Xi'an Jiaotong University Xi'an Shaanxi P.R. China

**Keywords:** all‐cause mortality, cardiovascular disease, depression, mediating effect

## Abstract

**Background:**

Cardiovascular disease (CVD) patients are more likely to have depression than general populations, and meanwhile, depression increased all‐cause mortality. However, the interaction effect of depression on CVD and all‐cause mortality has not been reported yet.

**Hypothesis:**

Herein, we speculate that depression may play an intermediate role in the association of CVD and all‐cause mortality.

**Methods:**

Demographic and clinical data of 33,156 adults (≥20 years old) were extracted from the National Health and Nutrition Examination Survey (NHANES) database in 2005−2018 in this retrospective cohort study. Weighted univariate and multivariate COX regression analyses were used to screen the covariates and to explore the relationship of CVD and depression. Distribution‐of‐product method was used to assess the mediating effect of depression on the association between CVD and all‐cause mortality. The mediating effect of depression was also explored in age, gender, diabetes mellitus (DM), and dyslipidemia subgroups. The evaluation indexes were odds ratios (ORs), hazard ratios (HRs), and 95% confidence intervals (CIs).

**Results:**

Among the participants, 11 514 had CVD, 5844 had depression, and 4759 were died. After adjusting for covariates, CVD was related to high odds of depression (OR = 1.94). Depression played an intermediate role in CVD and all‐cause mortality (HR = 1.23) with a mediational percentage of 9.13%. Subgroup analyses also showed this mediating effect existed in adults of different age, gender, DM and dyslipidemia status (all *p* < .05).

**Conclusion:**

The intermediate effect of depression may help clinicians to early identify high‐risk populations and provide some reference for disease management and mortality reduction.

## Graphical abstract

In this retrospective cohort study, we explored the mediating effect of depression on the relationship of cardiovascular disease (CVD) and all‐cause mortality. The results showed that CVD was related to high odds of depression (odds ratio = 1.94), and depression played an intermediate role in the association between CVD and the risk of all‐cause mortality (hazard ratios = 1.23, mediational percentage = 9.13%). Subgroup analyses showed that in adults of different age, sexes, having diabetes mellitus or not, and having dyslipidemia, depression also had the mediating effect (all *p* < .05).

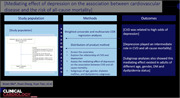

## 1 INTRODUCTION

Cardiovascular disease (CVD) is the leading cause of morbidity and mortality all over the world,^1^ and approximately accounts for one‐third of all deaths.^2^ Despite a global decline in age‐standardized CVD‐specific mortality over the past 30 years, the largest number of deaths from CVD occurred in China, followed by India, Russia, and the United States.^2^ Therefore, appropriate interventions and disease management for patients with CVD are important to reduce the disease burden.

The association between psychosocial factors and CVDs has long been recognized.^3^ Depression is a psychiatric condition and along with CVD currently represent the most common causes of disability in high‐income countries.^4^ The prevalence of depression increases with the severity of CVD and can predict cardiovascular morbidity and mortality.^5^ However, the vast majority of studies are not able to determine whether this association is causative or temporally related. Increasing evidences indicated that patients with CVD suffered from depression more than the general population.^6^, ^7^ A study in an elderly treated hypertensive population observed the incidence rate of depression was 4% per annum, and both all‐cause and CVD‐specific mortality of depression patients were increased compared to those without depression.^8^ Another cohort study in older men showed that although CVD as well as depression increased the risk of mortality, no interaction effect between them was observed.^9^ A previous study showed that of the total effect of subclinical CVD on all‐cause mortality in old persons, 16.3% was mediated by senile depression.^10^ Herein, we speculate that depression may play an intermediate role in the association of CVD and the risk of all‐cause mortality.

This study aims to explore the mediating effect of depression on the association between CVD and all‐cause mortality, and further discuss this mediating effect in age, gender, diabetes mellitus (DM), and dyslipidemia subgroups. It is hoped that this study to help early identify the high‐risk population in clinical, and provide some reference for the disease management and reducing the risk of morality.

## 2 METHODS

### 2.1 Study design and population

Data in this retrospective cohort study were extracted from the National Health and Nutrition Examination Survey (NHANES) database from 2005 to 2018. NHANES is a multipurpose research program done by the National Center for Health Statistics (NCHS) to assess the health and nutritional status of adults and children in the United States (https://www.cdc.gov/nchs/nhanes/index.htm). The survey regularly collects data of approximately 5000 persons from 15 areas since 1999 that includes a household interview followed by a standardized physical examination in a mobile examination center (MEC). A stratified multistage sampling design with a weighting scheme based on the selection of counties, blocks, households, and persons within households is used by NHANES to represent the civilian, noninstitutionalized US population and accurately estimate disease prevalence.

A total of 39 749 individuals aged ≥ 20 years old received the assessment of CVD and depression were initially included. Those who without information of marital status, body mass index (BMI), circumference, and survival were excluded, and finally, 33 156 of them were eligible. Since NHANES was approved by the Institutional Review Board (IRB) of the NCHS of the United States Centers for Disease Control and Prevention (CDC) and the data were publicly available, no ethical approval of our IRB was required.

### 2.2 Assessment of CVD and depression

The self‐reported NHANES multiple choice question (MCQ) was used to assess CVD: “Have you ever been told you had (congestive) heart failure, coronary heart disease, angina/angina pectoris, heart attack, or stroke.” Individuals who have a positive answer to the MCQ or taking cardiovascular drugs (the ID is 40 in RXQ_DRUG) were recognized as patients with CVD.

The patient health questionnaire‐9 (PHQ‐9) was administered during the face‐to‐face MEC interview to assess depressive symptoms over the last 2 weeks. The PHQ‐9 is validated as a depressive symptom severity measure (total score 1−4: minimal depression, 5−9: mild depression, 10−14: moderate depression, 15−19: moderately severe depression, and 20−27: severe depression), and total score ≥ 10 represents clinically significant depressive symptoms.^11^

### 2.3 Outcome and follow‐up

The study outcome was all‐cause mortality. The study data were extracted from NHANES from 2005 to 2018, and information of deaths were obtained through the National Death Index (NDI) to 31 December 2019.^12^ The follow‐up ended until patients' death during 2005−2018 or at the December 31, 2019.

### 2.4 Potential confounders

We extracted demographic and clinical information including age, gender, race, education level, marital status, poverty‐to‐income ratio (PIR), smoking and drinking status, physical activity, hypertension, DM, dyslipidemia, family history of CVD, BMI, circumference, total energy intake, med score, and estimated glomerular filtration rate (eGFR) of participants from the database.

In NHANES, all lifestyle factors were obtained through structured questionnaires and two 24‐hour dietary recalls. Never smoking was defined in the questionnaire as smoking fewer than 100 cigarettes in life. Frequency of current alcohol consumption was self‐reported (≤2 times a week or >2 times a week). Weekly metabolic equivalent (MET) hours of leisure time physical activity were calculated and converted to energy expenditure: energy expenditure (MET·minute) = recommended MET × exercise time of corresponding activity (minute). DM was assessed by laboratory examination (fasting blood glucose ≥ 7.0 mmol/L or HbAlc ≥ 6.5%), self‐report, or receiving hypoglycemic therapy. Dyslipidemia refers to total cholesterol ≥ 200 mg/dL (5.2 mmol/L) or triglyceride (TG) ≥ 150 mg/dL (1.7 mmol/L) or low‐density lipoprotein cholesterol (LDL‐C) ≥ 130 mg/dL (3.4 mmol/L) or high‐density lipoproteincholesterol (HDL‐C) ≤ 40 mg/dL (1.0 mmol/L) or self‐reported hypercholesterolemia or receiving lipid‐lowering therapy. eGFR was estimated based on serum creatinine: eGFR (mL/min/1.73 m^2^) = 141 × minute (Scr/κ, 1) *α* × max (Scr/κ, 1)−1.029 × 0.993 age × 1.108 (if female), κ is 0.7 for females and 0.9 for males, *α* is −0.329 for females and −0.411 for males, minute indicates the minimum of Scr/κ (mg/dL) or 1, and max indicates the maximum of Scr/κ (mg/dL) or 1. The MED score (total score = 18) are derived by an assigned value of “0,” “1,” or “2” across nine food categories (vegetables, legumes, fruits, nuts, whole grains, red and processed meats, fish, alcohol and olive oil), with higher scores indicating better adherence to MED pattern.^13^, ^14^ Hypertension was defined by laboratory examination (systolic blood pressure ≥ 140 mmHg or diastolic blood pressure ≥ 90 mmHg), self‐report, or taking blood pressure medication.

### 2.5 Statistical analysis

Quantitative data were described by mean ± standard error (mean ± SE), and t test was used for comparation. Categorical data were expressed as number with constituent ratio (*N* [%]), and chi‐square test for the comparison. Special sample weights are required to estimate more representative measures for general US population. WTSA2YR‐Two‐year (A subsample weights) included masked variance pseudo‐stratum, masked variance pseudo‐PSU and full sample MEC exam weight. More details can be obtained on the website: https://wwwn.cdc.gov/Nchs/Nhanes/2009-2010/UHM_F.htm#WTSA2YR.

Weighted univariate COX regression analysis was used to screen the covariates that associated with all‐cause mortality (Supporting Information: Table S1) and potential confounding factors were further screened by the weighted stepwise regression analysis. Weighted univariate and multivariate COX regression analyses were used to explore the association of CVD and depression with the evaluation index of odds ratios (OR) with 95% confidence intervals (CIs). COX regression model as a semiparametric model, with survival outcome and survival time as dependent variables, can simultaneously analyze the influence of many factors on survival time, and can analyze data with truncated survival time without requiring estimation of the survival distribution type of data.^15^ Model 1^[a]^ was the crude model. Model 2^[a]^ was adjusted for age, gender, race, smoking status, physical activity, DM, dyslipidemia, circumference, and eGFR.

We used distribution‐of‐product^16^ to explore the mediating effect of depression on the association between CVD and the risk of all‐cause mortality. Product distribution, indirect effect, mediational percentage, and 95% CIs were used to reflect the mediating effect. Model 1^[b]^ was adjusted for age, gender, race, smoking status, physical activity, DM, dyslipidemia, circumference, and eGFR. Model 2^[b]^ was adjusted for age, gender, race, smoking status, physical activity, DM, dyslipidemia, circumference, eGFR, and depression.

The interaction effect of CVD and depression on all‐cause mortality was explored using weighted univariate and multivariate COX regression analyses with hazard ratios (HRs) and 95%CIs. Model 1^[c]^ was the crude model. Model 2^[c]^ was adjusted for age, gender, race, smoking status, physical activity, DM, dyslipidemia, circumference, and eGFR.

We also explored these relationships in subgroups of age, gender, DM, and dyslipidemia. Two‐sided *p* < .05 was considered significant. Statistical analysis was performed using SAS 9.4 (SAS Institute) and R version 4.0.3 (Institute for Statistics and Mathematics). Missing variables including PIR and physical activity were classified as “unknown,” while others (education level, family history of CVD, total energy intake, smoking and drinking status and eGFR) were interpolated using random forest, and results of the sensitivity analysis were showed in Supporting Informtion: Table S2.

## 3 RESULTS

### 3.1 Characteristics of study population

**Figure 1:**
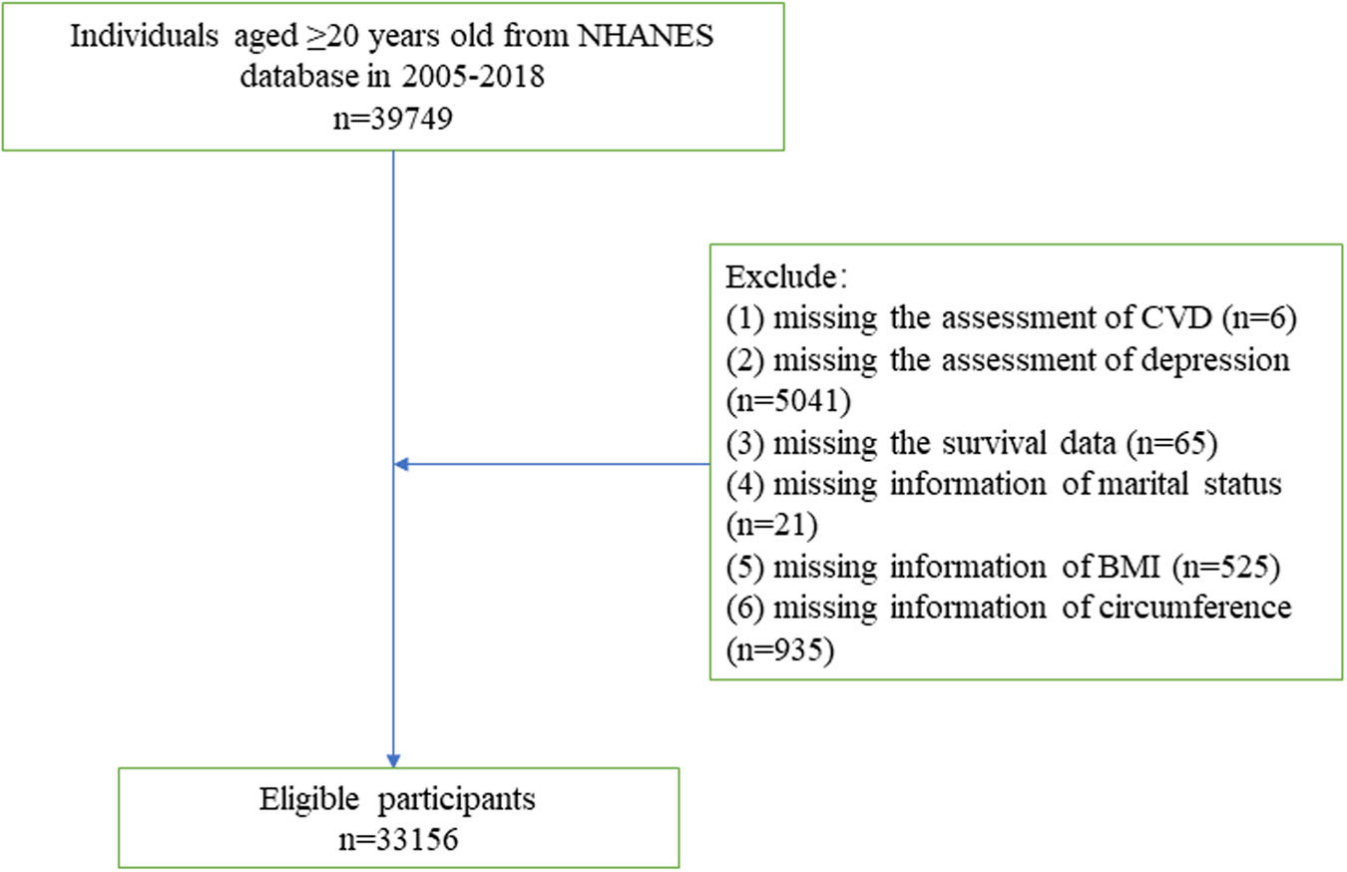
Flow chart of the participants screening.

Figure 1 is the flow chart of the participants screening. A total of 39 749 individuals aged ≥ 20 years old were initially included. Then we excluded those who without the survival data (*n* = 65), information of CVD (*n* = 6), depression (*n* = 5041), marital status (*n* = 21), BMI (*n* = 525) and circumference (*n* = 935). Finally, 33 156 of them were eligible.

The characteristics of the participants are showed in Table 1. The average follow‐up time was 101.97 months. Of the eligible individuals, 4759 (14.35%) were died, 11 514 (30.07%) had CVD, and 5844 (18.80%) had depression. The average age was 45.90 and 57.60 years old in survival group and mortality group, respectively. In survival group, 4501 (11.95%) adults had DM, and the number that in mortality group was 1293 (21.66%). Most of the participants in survival (19 325 [67.51%]) and mortality (3679 [76.21%]) group had dyslipidemia. Additionally, gender, race, education level, marital status, smoking status, physical activity, hypertension, family history of CVD, BMI, circumference, total energy intake, med score and eGFR were significantly different between the two groups (all *p* < .05).

**Table 1:** Characteristics of adults with and without CVD.

Variables	Total (*n* = 33 156)	Survival (*n* = 28 397)	All‐cause mortality (*n* = 4759)	Statistics	*p*
Age, years, Mean (SE)	47.27 (0.23)	45.90 (0.22)	57.60 (0.48)	*t* = −26.03	<.001
Gender, *n* (%)				*χ* ^2^ = 6.01	.014
Male	16 268 (48.73)	13 671 (48.44)	2597 (50.91)		
Female	16 888 (51.27)	14 726 (51.56)	2162 (49.09)		
Race, *n* (%)				*χ* ^2^ = 74.38	<.001
Mexican American	5208 (8.37)	4644 (8.76)	564 (5.43)		
Other Hispanic	3176 (5.47)	2876 (5.79)	300 (3.05)		
Non‐Hispanic White	14 220 (68.04)	11 521 (67.03)	2699 (75.66)		
Non‐Hispanic Black	7064 (10.90)	6073 (10.89)	991 (10.96)		
Other race—including multi‐Racial	3488 (7.22)	3283 (7.53)	205 (4.91)		
Education level, *n* (%)				*χ* ^2^ = 149.13	<.001
Less than 9th grade	3309 (5.03)	2632 (4.56)	677 (8.57)		
9‐11th grade (Includes 12th grade with no diploma)	4686 (10.40)	3825 (9.88)	861 (14.23)		
High school graduate/GED or equivalent	7664 (23.34)	6430 (22.87)	1234 (26.83)		
Some college or AA degree	9878 (31.67)	8683 (32.03)	1195 (28.95)		
College graduate or above	7619 (29.57)	6827 (30.65)	792 (21.42)		
Marital status, *n* (%)				*χ* ^2^ = 529.30	<.001
Married	17150 (55.61)	14 777 (55.94)	2373 (53.13)		
Widowed	2493 (5.45)	1626 (4.21)	867 (14.81)		
Divorced	3635 (10.36)	3039 (10.13)	596 (12.03)		
Separated	1123 (2.41)	956 (2.37)	167 (2.70)		
Never married	6005 (17.88)	5480 (18.66)	525 (12.04)		
Living with partner	2750 (8.29)	2519 (8.69)	231 (5.28)		
PIR, *n* (%)				*χ* ^2^ = 3.66	.160
<1.0	6351 (12.92)	5423 (12.76)	928 (14.07)		
≥1.0	24084 (80.52)	20 584 (80.60)	3500 (79.87)		
Unknown	2721 (6.57)	2390 (6.63)	331 (6.06)		
Drinking status, *n* (%)				*χ* ^2^ = 0.00	.994
Less than twice a week (including 2)	28616 (83.14)	24 571 (83.14)	4045 (83.15)		
More than twice a week	4540 (16.86)	3826 (16.86)	714 (16.85)		
Smoking status, *n* (%)				*χ* ^2^ = 119.89	<.001
No	18201 (54.72)	16 170 (56.27)	2031 (43.11)		
Yes	14 955 (45.28)	12 227 (43.73)	2728 (56.89)		
Physical activity, MET·min/week, *n* (%)				*χ* ^2^ = 414.76	<.001
<450	3656 (10.82)	3026 (10.48)	630 (13.38)		
≥450	20402 (66.27)	18 267 (68.49)	2135 (49.61)		
Unknown	9098 (22.92)	7104 (21.04)	1994 (37.01)		
Hypertension, *n* (%)				*χ* ^2^ = 352.87	<.001
No	19060 (62.24)	17187 (64.47)	1873 (45.52)		
Yes	14096 (37.76)	11 210 (35.53)	2886 (54.48)		
DM, *n* (%)				*χ* ^2^ = 302.35	<.001
No	27362 (86.90)	23 896 (88.05)	3466 (78.34)		
Yes	5794 (13.10)	4501 (11.95)	1293 (21.66)		
Dyslipidemia, *n* (%)				*χ* ^2^ = 72.09	<.001
No	10152 (31.46)	9072 (32.49)	1080 (23.79)		
Yes	23004 (68.54)	19325 (67.51)	3679 (76.21)		
Family history of CVD, *n* (%)				*χ* ^2^ = 23.36	<.001
No	28 963 (86.65)	24 898 (87.02)	4065 (83.87)		
Yes	4193 (13.35)	3499 (12.98)	694 (16.13)		
BMI, kg/m^2^, Mean (S.E)	29.02 (0.08)	29.05 (0.09)	28.78 (0.11)	*t* = 2.06	.042
Circumference, cm, Mean (S.E)	99.31 (0.22)	99.12 (0.23)	100.74 (0.32)	*t* = −4.60	<.001
Total energy intake, kcal, Mean (S.E)	2189.29 (8.33)	2207.76 (7.93)	2050.86 (23.88)	*t* = 6.86	<.001
Med score, Mean (SE)	4.16 (0.03)	4.17 (0.03)	4.06 (0.05)	*t* = 2.28	.025
eGFR, mL/min/1.73 m^2^, Mean (SE)	103.22 (0.28)	104.82 (0.28)	91.20 (0.65)	*t* = 21.67	<.001
Follow‐up time, months, Mean (SE)	101.97 (1.37)	101.74 (1.47)	103.69 (1.75)	*t* = −0.99	.324
CVD, *n* (%)				*χ* ^2^ = 596.61	<.001
No	21642 (69.93)	19619 (72.61)	2023 (49.82)		
Yes	11514 (30.07)	8778 (27.39)	2736 (50.18)		
Depression, *n* (%)				*χ* ^2^ = 26.71	<.001
No	27312 (81.20)	23 593 (81.72)	3719 (77.26)		
Yes	5844 (18.80)	4804 (18.28)	1040 (22.74)		

Abbreviations: χ^2^, chi‐square test; BMI, body mass index; CVD, cardiovascular disease; DM, diabetes mellitus; eGFR, estimated glomerularfiltrationrate; MET, metabolic equivalent of energy; PIR, poverty‐income ratio; SE, standard error, t, test.

### 3.2 Relationship between CVD and depression, and in subgroups of age, gender, DM and dyslipidemia

The association between CVD and depression and in different subgroups is showed in Table 2. After adjusting for the covariates, CVD was related to high odds of depression [OR = 1.94, 95%CI: (1.77−2.13)]. This relationship was also found in individuals aged < 65 years old (OR = 1.94), ≥65 years old (OR = 1.94), male adults (OR = 1.94), female adults (OR = 1.94), participants who had DM (OR = 1.94) or not have DM (OR = 1.94), had dyslipidemia (OR = 1.94) or not have dyslipidemia (OR = 1.94).

**Table 2:** Association between CVD and depression, and in subgroups of age, gender, DM and dyslipidemia.

Subgroups	Model 1^[a]^		Model 2^[a]^	
	OR (95% CI)	*p*	OR (95% CI)	*p*
Total individuals	2.20 (2.04−2.36)	<.001	1.94 (1.77−2.13)	<.001
Age <65	2.55 (2.32−2.79)	<.001	2.01 (1.80−2.25)	<.001
Age ≥65	1.86 (1.56−2.22)	<.001	1.75 (1.46−2.09)	<.001
Male	2.35 (2.10−2.63)	<.001	2.23 (1.93−2.57)	<.001
Female	2.13 (1.95−2.33)	<.001	1.83 (1.63−2.06)	<.001
DM	1.89 (1.53−2.33)	<.001	2.06 (1.64−2.59)	<.001
Non‐DM	2.17 (1.99−2.37)	<.001	1.92 (1.72−2.14)	<.001
Dyslipidemia	2.02 (1.85−2.20)	<.001	1.96 (1.76−2.18)	<.001
Nondyslipidemia	2.37 (1.98−2.83)	<.001	1.94 (1.55−2.43)	<.001

*Note*: Model 1^[a]^: crude model; Model 2^[a]^: adjusted for age, gender, race, smoking status, physical activity, DM, dyslipidemia, circumference, and eGFR.

Abbreviations: CI, confidence interval; CVD, cardiovascular disease; DM, diabetes mellitus; eGFR, estimated glomerularfiltrationrate; OR, odds ratio.

### 3.3 Mediating effect of depression on the AAO of CVD and all‐cause mortality, and in subgroups of age, gender, DM and dyslipidemia

**Figure 2:**
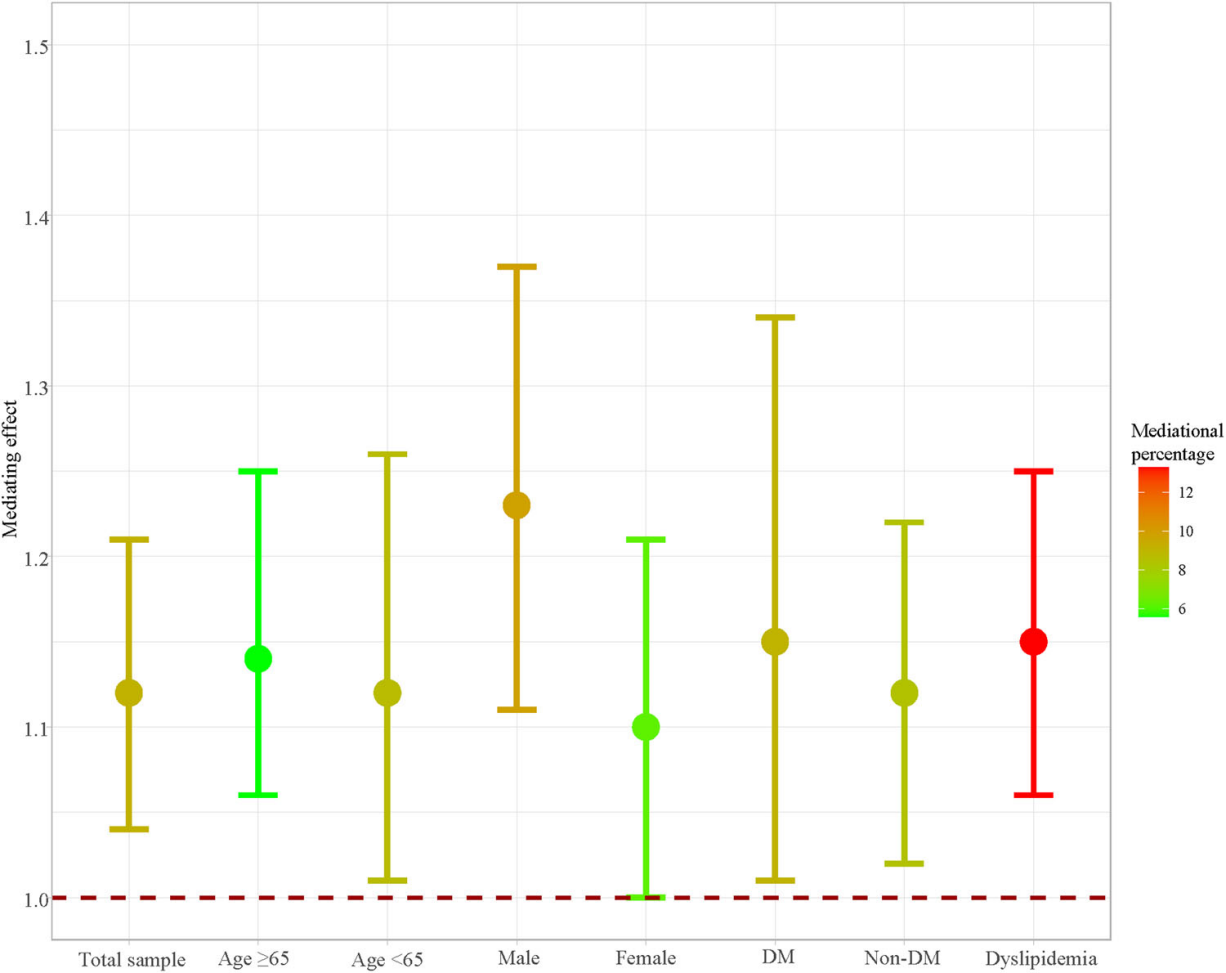
The mediating effect of depression on the association between CVD and all‐cause mortality in aged, gender, DM and dyslipidemia subgroups. CVD, cardiovascular disease; DM, diabetes mellitus.

Table 3 shows the mediating effect of depression on correlation between CVD and the risk of all‐cause mortality, and in different subgroups. We found that depression may play a mediating role in the CVD and the risk of all‐cause mortality (HR = 1.23, 95% CI: [1.13−1.34]), with a product distribution of 0.11 with 95% CI of 0.04−0.19, and the mediational percentage was 9.13%. Furthermore, the mediating effect of depression was also observed in individuals of different age, gender, with/without DM, and with dyslipidemia (all 95% CI were excluded “0”) (Figure 2).

**Table 3:** Mediating effect of depression on the association between CVD and the risk of all‐cause mortality, and in subgroups of age, gender, DM and dyslipidemia.

Subgroups	Model 1^[b]^	Model 2^[b]^	Product distribution (95% CI)	Indirect Effect (95% CI)	Mediational percentage (%)
	HR (95% CI)	HR (95% CI)			
Total individuals	1.25 (1.14−1.37)	1.23 (1.13−1.34)	0.11 (0.04−0.19)	1.12 (1.04−1.21)	9.13
Age ≥65	1.38 (1.23−1.54)	1.36 (1.21−1.51)	0.13 (0.06−0.22)	1.14 (1.06−1.25)	5.57
Age <65	1.32 (1.15−1.52)	1.29 (1.13, 1.49)	0.12 (0.01−0.23)	1.12 (1.01−1.26)	8.70
Male	1.36 (1.18−1.57)	1.32 (1.14−1.53)	0.20 (0.10−0.31)	1.23 (1.11−1.37)	9.80
Female	1.37 (1.20−1.56)	1.35 (1.18−1.53)	0.09 (0.00−0.19)	1.10 (1.00−1.21)	6.23
DM	1.34 (1.06−1.70)	1.31 (1.04−1.66)	0.14 (0.01−0.29)	1.15 (1.01−1.34)	9.04
Non‐DM	1.25 (1.14−1.37)	1.23 (1.12−1.35)	0.11 (0.02−0.20)	1.12 (1.02−1.22)	8.47
Dyslipidemia	1.20 (1.09−1.33)	1.18 (1.07−1.29)	0.14 (0.05−0.22)	1.15 (1.06−1.25)	13.30
Non‐dyslipidemia	1.38 (1.10−1.74)	1.37 (1.09−1.72)	0.04 (−0.11 to 0.19)	1.04 (0.90−1.21)	

*Note*: Model 1^[b]^: adjusted for age, gender, race, smoking status, physical activity, DM, dyslipidemia, circumference, and eGFR; Model 2^[b]^: adjusted for age, gender, race, smoking status, physical activity, DM, dyslipidemia, circumference, eGFR, and depression.

Abbreviations: CI, confidence interval; CVD, cardiovascular disease; DM, diabetes mellitus; eGFR, estimated glomerularfiltrationrate; HR, hazard ratio; OR, odds ratio.

### 3.4 The interaction effect of CVD and depression on all‐cause mortality

We further explored the interaction effect of CVD and depression on all‐cause mortality (Table 4). After adjusted for covariates, there was no significant interaction effect of CVD and depression on all‐cause mortality (all *p* > .05), indicating a reliable mediating effect.

**Table 4:** The interaction effect of CVD and depression on all‐cause mortality.

Subgroups	Variables	Model 1^[c]^		Model 2^[c]^	
		HR (95% CI)	*p*	HR (95% CI)	*p*
Total individuals	CVD	2.72 (2.52−2.94)	<.001	1.18 (1.08−1.30)	.001
	Depression	1.15 (0.97−1.36)	.110	1.07 (0.91−1.27)	.409
	CVD * Depression	1.00 (0.82−1.20)	.964	1.18 (0.97−1.45)	.103
Age ≥ 65	CVD	1.58 (1.42−1.76)	<.001	1.33 (1.18−1.49)	<.001
	Depression	1.09 (0.74−1.60)	.655	1.14 (0.79−1.64)	.497
	CVD * Depression	1.23 (0.81−1.86)	.331	1.14 (0.75−1.73)	0.531
Age < 65	CVD	1.57 (1.38−1.79)	<.001	1.25 (1.07−1.45)	.004
	Depression	1.19 (0.98−1.45)	.086	1.12 (0.92−1.37)	.253
	CVD * Depression	1.14 (0.87−1.50)	.345	1.14 (0.85−1.52)	.382
Male	CVD	2.81 (2.50−3.15)	<.001	1.31 (1.13−1.51)	<.001
	Depression	1.32 (1.05−1.67)	.018	1.24 (0.99−1.56)	.062
	CVD * Depression	0.97 (0.72−1.31)	.837	1.06 (0.79−1.43)	.681
Female	CVD	2.63 (2.31−2.98)	<.001	1.28 (1.11−1.47)	.001
	Depression	1.09 (0.89−1.34)	.396	1.05 (0.85−1.29)	.649
	CVD * Depression	1.02 (0.81−1.29)	.857	1.20 (0.94−1.54)	.145
DM	CVD	2.40 (1.92−2.99)	<.001	1.20 (0.93−1.54)	.160
	Depression	0.83 (0.53−1.30)	.411	0.81 (0.49−1.35)	.419
	CVD * Depression	1.44 (0.89−2.32)	.135	1.57 (0.93−2.66)	.093
Non‐DM	CVD	2.46 (2.24−2.71)	<.001	1.19 (1.08−1.33)	.001
	Depression	1.17 (0.98−1.40)	.086	1.12 (0.93−1.33)	.234
	CVD * Depression	0.94 (0.76−1.16)	.550	1.13 (0.91−1.41)	.282
Dyslipidemia	CVD	2.59 (2.37−2.84)	<.001	1.15 (1.03−1.27)	.011
	Depression	1.14 (0.95−1.38)	.169	1.13 (0.93−1.38)	.219
	CVD * Depression	1.03 (0.83−1.28)	.808	1.13 (0.90−1.42)	.295
Non‐dyslipidemia	CVD	2.94 (2.49−3.48)	<.001	1.32 (1.03−1.69)	.029
	Depression	1.13 (0.84−1.51)	.428	1.00 (0.73−1.35)	.977
	CVD * Depression	0.84 (0.57−1.24)	.387	1.17 (0.77−1.78)	.453

*Note*: Model 1^[c]^: crude model., Model 2^[c]^: adjusted for age, gender, race, smoking status, physical activity, DM, dyslipidemia, circumference, and eGFR.

Abbreviations: CI, confidence interval; CVD, cardiovascular disease; DM, diabetes mellitus; eGFR, estimated glomerularfiltrationrate; HR, hazard ratio; OR, odds ratio.

## 4 DISCUSSION

In this study, we explored the mediating effect of depression on the relationship of CVD and all‐cause mortality. The results showed that CVD was related to high odds of depression, and depression played an intermediate role in the correlation between CVD and the risk of all‐cause mortality. Subgroup analyses showed that in adults of different age, sexes, having DM or not, and having dyslipidemia, depression also had the mediating effect.

A gap in the literature exists in the mediating effect of depression on the association between CVD and all‐cause mortality in adults. Armstrong et al.^10^ evaluated whether late‐life depression mediates the association of subclinical CVD with all‐cause mortality and found that late‐life depression explained 16.3% of the total effect of subclinical CVD on the risk of all‐cause mortality. In the current study, we discovered a mediating effect of depression on correlation of CVD and all‐cause mortality with a mediational percentage of 9.13%. Cardiovascular events may increase the risk of depression.^17^ The biological link between depression and CVD may be related to the overactivation of the hypothalamic−pituitary−adrenal axis results in the dysregulation of immune system and further causes high levels of proinflammatory cytokines (such as IL‐1β, TNF‐α, and IL‐6) released by macrophages.^18^ Depression and its symptoms are associated with increased risk of all‐cause and CVD‐specific mortality.^19^ Previous studies had proposed several potential causal mechanisms for association between depression and mortality but there is no consensus yet.^20^, ^21^ Depression may cause dysregulation of central biological stress systems, such as hypothalamic−pituitary−adrenal axis hyperactivity,^22^ neuroimmune and sympathoadrenergic dysregulation.^23^ Additionally, people with depression usually have unhealthy lifestyles, including smoking, heavy alcohol consumption, and low adherence to treatment, that have been consistently shown to be causal risk factors for premature death.^21^ Based on this, we suggested that the potential mechanism of the mediating role of depression in the relationship of CVD and all‐cause mortality may be through an increased inflammatory state involving by the hypothalamic−pituitary−adrenal axis that leads to central system disorders and further affects the risk of death.

We also explored this mediating effect in age, gender, DM and dyslipidemia subgroups, and found that depression played a mediating role in the association between CVD and all‐cause mortality in adults aged ≥ 65 or <65 years old, male or female, having DM or not, and having dyslipidemia. A previous study has found a stronger association between depression and stroke in individuals aged < 65 years old but not in those ≥65 years old.^24^ More prospective studies with a large sample size are calling to confirm whether age modifies the association between depression and CVD and mortality in the future. Depression is more prevalent in women than men, nearly of a doubled chance.^25^ Indeed, it seems that all the phases of women's life, characterized by greatest hormonal changes and inherent modulations of estrogen and progesterone levels, are affected and influenced by both biological and psychosocial factors.^26^ For a long time, the annual CVD‐specific mortality rate has remained greater for female than male.^27^ The occurrence of depression can worsen CVD morbidity and mortality in women.^4^ Since the findings on the relationship between depression and CVD risk by sex are not consistent among studies,^28^, ^29^ and we similarly not find any difference of the mediating effect of depression on CVD and all‐cause mortality between women and men, more basic researched are required to explore the potential mechanism for sex‐related factors affecting depression's mediating influence. Melin et al.^30^ found that in patients with newly diagnosed type 2 diabetes (T2D), the younger women had the highest prevalence of depression, which was the risk factor for CVD. There mechanisms that could explain the association between depression and CVD events in patients with T2D induce the following alterations of autonomic nervous system activity, decrease of heart‐rate variability, the elevation of heart rate, catecholamine levels, and inflammatory activity, and the induction of endothelial and platelet dysfunction.^31^, ^32^ Similar to the previous study by Kim et al.^33^ founding that dyslipidemia patients with pre‐existing depression had increased risk for CVD. Future studies that determine all‐cause mortality risk after management of depression among CVD patients with dyslipidemia are needed.

Data in this study were extracted from the NHANES database, which using a multistage sampling design with a weighting scheme that the sample size was large and representative. We found the mediating effect of depression on the association between CVD and all‐cause mortality that partly provide some reference for reduction and disease management of CVD. However, there are still some limitations in this study. This study is a retrospective study so that CVD and depression status were assessed at the same time point, and their changes during follow‐up were not available. In addition, other possible confounding factors such as treatment and lifestyle during follow‐up were difficult to take into account.

## 5 CONCLUSION

Depression plays an intermediate role in the association between CVD and all‐cause mortality, indicating that it may help to early identify the high‐risk population, and provide some reference for the disease management and reducing the risk of morality.

## CONFLICT OF INTEREST STATEMENT

The authors declare no conflict of interest.

## Supporting information

Supporting information.

## Data Availability

The datasets used and/or analyzed during the current. study are available from the corresponding author on reasonable request.
