# Supplementary material for: Mediating effect of depression on the association between cardiovascular disease and the risk of all‐cause mortality: NHANES in 2005−2018
Source: Clin Cardiol. 2023 Aug 18;46(11):1380–9. doi: 10.1002/clc.24103 (PMC10642320; doi:10.1002/clc.24103)
Supplement: Supplementary file 1 — Supporting information. [file CLC-46-1380-s001.docx]

**Table S1. Confounding factors of all-cause mortality**

| Variables | HR (95% CI) | *P* |
| --- | --- | --- |
| CVD |  |  |
| No | Ref |  |
| Yes | 2.77 (2.56-2.99) | <0.001 |
| Depression |  |  |
| No | Ref |  |
| Yes | 1.37 (1.24-1.53) | <0.001 |
| Age | 1.05 (1.04-1.05) | <0.001 |
| Gender |  |  |
| Male | Ref |  |
| Female | 0.89 (0.83-0.96) | 0.002 |
| Race |  |  |
| Mexican American | Ref |  |
| Other Hispanic | 0.97 (0.77-1.22) | 0.778 |
| Non-Hispanic White | 1.72 (1.51-1.97) | <0.001 |
| Non-Hispanic Black | 1.56 (1.34-1.80) | <0.001 |
| Other Race - Including Multi-Racial | 1.28 (1.04-1.59) | 0.023 |
| Education level |  |  |
| Less than 9th grade | Ref |  |
| 9-11th grade (Includes 12th grade with no diploma) | 0.77 (0.67-0.89) | <0.001 |
| High school graduate/GED or equivalent | 0.70 (0.61-0.81) | <0.001 |
| Some college or AA degree | 0.56 (0.48-0.65) | <0.001 |
| College graduate or above | 0.45 (0.39-0.53) | <0.001 |
| Marital status |  |  |
| Married | Ref |  |
| Widowed | 3.51 (3.21-3.83) | <0.001 |
| Divorced | 1.26 (1.11-1.44) | <0.001 |
| Separated | 1.22 (0.99-1.52) | 0.064 |
| Never married | 0.75 (0.66-0.85) | <0.001 |
| Living with partner | 0.68 (0.56-0.83) | <0.001 |
| PIR |  |  |
| <1.0 | Ref |  |
| ≥1.0 | 0.85 (0.76-0.96) | 0.011 |
| Unknown | 0.97 (0.81-1.16) | 0.711 |
| Drinking status |  |  |
| Less than twice a week (including 2) | Ref |  |
| More than twice a week | 1.00 (0.88-1.13) | 0.967 |
| Smoking status |  |  |
| No | Ref |  |
| Yes | 1.56 (1.43-1.70) | <0.001 |
| Physical activity |  |  |
| <450 | Ref |  |
| ≥450 | 0.75 (0.67-0.84) | <0.001 |
| Unknown | 1.49 (1.33-1.66) | <0.001 |
| Hypertension |  |  |
| No | Ref |  |
| Yes | 2.24 (2.07-2.43) | <0.001 |
| DM |  |  |
| No | Ref |  |
| Yes | 2.32 (2.13-2.52) | <0.001 |
| Dyslipidemia |  |  |
| No | Ref |  |
| Yes | 1.45 (1.32-1.59) | <0.001 |
| Family history of CVD |  |  |
| No | Ref |  |
| Yes | 1.21 (1.09-1.34) | <0.001 |
| BMI | 1.00 (1.00-1.01) | 0.431 |
| Circumference | 1.01 (1.01-1.01) | <0.001 |
| Total energy intake | 1.00 (1.00-1.00) | <0.001 |
| Med score | 1.00 (0.98-1.01) | 0.568 |
| eGFR | 0.97 (0.97-0.98) | <0.001 |

CVD: cardiovascular disease, HR: hazard ratio, CI: confidence interval, PIR: poverty-income ratio, DM: diabetes mellitus, BMI: body mass index, eGFR: estimated glomerularfiltrationrate

**Table S2. Sensitivity analysis of characteristic of participants before and after interpolation**

| Variables | Before interpolation | After interpolation | Statistics | *P* |
| --- | --- | --- | --- | --- |
| Education level, n (%) |  |  | χ^2^=2.89 | 0.576 |
| Less than 9th grade | 3304 (5.03) | 3309 (5.03) |  |  |
| 9-11th grade (Includes 12th grade with no diploma) | 4682 (10.40) | 4686 (10.40) |  |  |
| High school graduate/GED or equivalent | 7657 (23.34) | 7664 (23.34) |  |  |
| Some college or AA degree | 9872 (31.66) | 9878 (31.67) |  |  |
| College graduate or above | 7617 (29.57) | 7619 (29.57) |  |  |
| Drinking status, n (%) |  |  | χ^2^=1.37 | 0.242 |
| Less than twice a week (including 2) | 27973 (83.09) | 28616 (83.14) |  |  |
| More than twice a week | 4480 (16.91) | 4540 (16.86) |  |  |
| Smoking status, n (%) |  |  | χ^2^=0.03 | 0.864 |
| No | 18194 (54.72) | 18201 (54.72) |  |  |
| Yes | 14947 (45.28) | 14955 (45.28) |  |  |
| Family history of CVD, n (%) |  |  | χ^2^=2.01 | 0.156 |
| No | 28265 (86.71) | 28963 (86.65) |  |  |
| Yes | 4080 (13.29) | 4193 (13.35) |  |  |
| Total energy intake, kcal, Mean (S.E) | 2190.88 (8.50) | 2189.29 (8.33) | t=1.47 | 0.146 |
| eGFR, mL/min/1.73m^2^, Mean (S.E) | 103.17 (0.29) | 103.22 (0.28) | t=-1.78 | 0.077 |

t: test, χ2: chi-square test

CVD: cardiovascular disease, SE: standard error, eGFR: estimated glomerularfiltrationrate
